# Intratumoral Delivery of Genetically Engineered Anti-IL-6 Trans-signaling Therapeutics

**DOI:** 10.1007/s12033-024-01230-6

**Published:** 2024-07-09

**Authors:** Raphaela Bento, Jürgen Scheller, Biju Parekkadan

**Affiliations:** 1 Department of Biomedical Engineering, Rutgers University, Piscataway, NJ USA; 2 Institute of Biochemistry and Molecular Biology II, Medical Faculty, Heinrich-Heine-University, Düsseldorf, Germany

**Keywords:** Gene delivery, Genetically engineered cells, Cell-based delivery, Interleukin-6, Gene therapeutics, Anti-cytokine therapy, IL-6 trans-signaling

## Abstract

Interleukin-6 (IL-6) is a highly pro-inflammatory cytokine involved in the etiopathology of several inflammatory diseases and cancer. As so, the inhibition of IL-6 signaling pathways has emerged as an attractive therapeutic avenue for the treatment of several chronic diseases. Since IL-6 *trans*-signaling was described as the pathological branch of IL-6, selective inhibitors were developed. Next-generation variants with increased trans-signaling specificity and potency emerged as great candidates for the treatment of several diseases, with reduced off-target effects. The highly time-consuming and costly processes involving recombinant protein production, however, have hampered the progress of anti-cytokine pharmaceuticals in clinic so far. Herein, we developed gene therapeutic modalities of IL-6-trans-signaling inhibitors as alternatives for sustained recombinant protein secretion*.* By using an IL-6-dependent lymphoma cell line and xenograft tumor model, we demonstrated the superior inhibitory potential of second-generation anti-IL-6 trans-signaling therapeutic. We compared the efficiency of distinct gene delivery modalities using a bioluminescent biomarker probe and observed consistent protein production via cell-based delivery. When delivered intratumorally, genetically engineered sgp130^FlyR^Fc-secreting cells significantly reduced tumor burden and increased animal survival, representing a promising therapeutic avenue to be explored in clinically relevant gene delivery applications.

**Supplementary Information:**

The online version contains supplementary material available at 10.1007/s12033-024-01230-6.

## Introduction

Interleukin-6 (IL-6) is a key immunomodulatory cytokine involved in distinct physiological and pathophysiological functions, ranging from cell development and differentiation to inflammation and cancer, being heavily synthesized in the body during infections and inflammatory processes [1, 2]. Human IL-6 was one of the first cytokines to be molecularly defined, and it is now known to operate under three specific signaling modes: (a) classic signaling, that occurs when IL-6 binds to its membrane-bound receptor (mIL-6R) and to the signal-transducing homodimer of glycoprotein130 (gp130); (b) trans-signaling, when IL-6 forms a complex with the soluble version of human IL-6 receptor (sIL-6R)—generated either via proteolysis [3] or alternative splicing [4]—which activates the membrane-bound gp130; and (c) cluster signaling, when gp130 subunits are activated by IL-6/mIL-6R complexes formed in a neighboring transmitter cell [5]. All pathways involve the downstream activation of Janus kinase (JAK) and signal transducer and activator of transcription (STAT) signaling, resulting in multiple cellular and molecular responses. While IL-6 classic signaling has mostly been associated with protective homeostatic responses, sIL-6R-mediated trans-signaling has been described as the “pathological mode of IL-6 signaling” [2]. Hyper IL-6, a fusion protein of IL-6 and sIL-6R, was designed by Dr. Rose-John et al. and helped elucidate IL-6-trans-signaling-specific mechanisms and pro-inflammatory responses, such as recruitment of mononuclear cells, inhibition of T-cell apoptosis, and inhibition of Treg differentiation [2, 5, 6].

IL-6 inhibition emerged as an appealing therapeutic avenue in view of its clear association with the pathogenesis of many diseases, ranging from bacterial and viral infections [7] to arthritis [8], inflammatory bowel diseases (IBD) [9], and multiple sclerosis [5, 10, 11]. Humanized monoclonal antibodies targeting IL-6 or IL-6R were the first inhibitors to make their way into the clinic, and several are still being tested in pre-clinical studies and clinical trials [12]. Tocilizumab, a commercially available anti-IL-6R antibody, prevents binding of IL-6 to its receptor, globally inhibiting IL-6 signaling pathway [5, 13].

Once IL-6 trans-signaling was recognized as the pro-inflammatory arm of IL-6, a selective inhibitor was designed [14]. Soluble gp130Fc (sgp130Fc) is a fusion protein of the extracellular moiety of gp130 in combination with the fragment crystallizable region (Fc) of human IgG1 antibody. sgp130Fc blocks IL-6 trans-signaling by interacting with IL-6/sIL-6R complexes and preventing their binding to membrane-bound gp130, without interfering with classic signaling. Olamkicept is currently in phase IIb clinical trial for the treatment of IBD and results are promising so far [15]. The inhibitory activity of sgp130Fc, however, was later shown to extend to other members of the IL-6 cytokine family with similar potency, such as IL-11 [16, 17]. The solution was found in the rational design of next-generation variants, with higher specificity and affinity toward IL-6 trans-signaling. sgp130^FlyR^Fc variant contains three amino-acid substitutions at specific sites, resulting in more potent IL-6 trans-signaling inhibition and complete abrogation of IL-11 blockade [18]. The inhibitory potential of this variant has been described in vitro*,* though has yet to be tested in animal models or formulated for long-term drug delivery applications.

Intricate and costly processes involving recombinant protein and monoclonal antibody production and purification have hindered some of the progress in the anti-cytokine field thus far [19, 20]. The need for biologicals to treat inflammatory diseases and cancer is rising more than ever and targeted anti-cytokine therapy and precision medicine have been the focus of the scientific community in recent years, given that both areas can coevolve into effective treatments for patients in need [21–23]. Several chronic inflammatory diseases require long-term treatments with daily drug regimens, limiting patient adhesion and the use of such pharmaceuticals in clinic [24]. Time and cost-effective alternatives are much needed to circumvent these problems. Herein, we developed gene therapeutic forms of sgp130 variants and evaluated the anti-IL-6 potency using a human IL-6-dependent lymphoma cell line in cell culture and xenograft tumor models. We established a proof-of-concept framework for intratumoral delivery of anti-cytokine therapeutics by engrafting a genetically engineered cell line, that resulted in significant reduction of tumor burden and improved animal survival. This gene therapeutic approach is an alternative to recombinant protein production and systemic administration, thereby enabling a sustained delivery method of IL-6 trans-signaling blockade in situ.

## Materials and Methods

### Cell Culture

The generation of Ba/F3-gp130 and Ba/F3-gp130-IL-6R cells (generously provided by Dr. Jürgen Scheller and Dr. Jens Moll, Düsseldorf University) has been described elsewhere [25]. DS-1 cells (ATCC) and Ba/F3 cells were cultured in Dulbecco’s modified Eagle’s medium (DMEM) high-glucose culture medium (Thermo Fisher) supplemented with 10% FBS (Gibco, Life Technologies) and 1% antibiotic/antifungal v/v (Thermo Fisher). Proliferation of DS-1 cells was maintained in the presence of 10ng/mL of recombinant human IL-6 (R&D systems). Proliferation of Ba/F3-gp130 cells was maintained in the presence of 10ng/mL of Hyper IL-6 (provided by Dr. Scheller). Proliferation of Ba/F3-gp130-IL6R cells was maintained in the presence of 10ng/mL of IL-6. HEK293T embryonic kidney cells (ATCC) were cultured in DMEM F/12 medium (Thermo Fisher) supplemented with 10% FBS and 1% antibiotic/antifungal. Human mesenchymal stem cells (hMSCs) were cultured in RoosterNourish™-MSC medium (RoosterBio), supplemented with RoosterReplenish™-MSC-XF (RoosterBio). All cells were incubated in 5% CO_2_ at 37 °C.

### Viral Vector Production

#### Lentiviral Transfection

The LV-GFP-EF1a-sgp130Fc-IRES-GLuc plasmid was designed and purchased from VectorBuilder (plasmid ID: VB210721-1169kng). The plasmid containing sgp130^FlyR^Fc sequence was generously provided by Dr. Jürgen Scheller (Dusseldorf University) and the coding sequence was used to create the LV-GFP-EF1a-sgp130^Fly^^R^Fc plasmid. The packaging (pspax2) and envelope plasmids (pmd2g) were purchased from Addgene (plasmids #12,260 and #12,259, respectively). All plasmid stocks were maxi-prepped using HiPure Maxi column-based preparation kits (Invitrogen) and concentrations as well as purity ratios were recorded using a NanoDrop plate on VarioScan software (Invitrogen). Lentiviral vectors were generated using triple-transfection methods in adherent HEK293T cells. Cells were seeded in 15 cm dishes at 40% confluence the day before transfection. Plasmids were mixed at a 1:1:1 molar ratio with PEI and incubated with OptiMEM medium (Gibco) for 15 min. Cell culture medium was replaced with OptiMEM and DNA-PEI complexes were added dropwise. Transfection culture was carried out for 72 h until supernatant was collected and filtered through a 0.45 um PES membrane filter. Filtrate was further purified via sucrose gradient, upon ultracentrifugation at 26,000 rpm for 90 min at 4 °C. Lentiviral pellet was gently resuspended in sterile PBS and stored in − 80 °C freezer. Vector titer was determined by RT-qPCR using an LV titration kit (Applied Biologic Materials) on Quant Studio 3 (Thermo Fisher).

#### Lentiviral Transduction

3 × 10^4^ HEK293T cells were plated in 48-well plates and lentiviral vectors expressing either sgp130Fc or sgp130^FlyR^Fc were added at increasing multiplicity of infections (MOIs) (20, 30, 50, and 100 IU/cell), along with 1X ViralEntry™ Transduction Enhancer (Applied Biological Materials Inc.). After 72 h, transduction efficiency was assessed via GFP fluorescence using Celigo Imaging Cytometer (Nexcelom Bioscience) and GLuc or sgp130Fc concentration in the supernatant, as detailed below. Group with highest transduction efficiency (% GFP^+^) was expanded over the next week and GFP^+^ cells were sorted as described below. Sorted cells were expanded and a cell bank of engineered cells was created.

### Cell Sorting

Flow cytometry was performed by the Flow Cytometry & Confocal Microscopy Core Facility at Rutgers University. Transduced HEK cells were trypsinized and resuspended in FACS buffer. The mixed cell population was stained with DAPI for 5 min. Cell sorting was performed using a MoFlo Astrios EQ (Beckman Coulter, USA) sorter, equipped with 405 and 488 nm lasers for excitation, a 100 µm nozzle, and IsoFlow sheath fluid (Beckman Coulter, USA) maintained at 25 psi. Cells were deposited into coated 5-mL polypropylene tubes containing 3mL collection media. To identify and recover GFP^+^ DAPI^−^ cells, sort decisions were made by visualization of a bivariate plot of 488–513/26 Area log (GFP) and 405–448–448/59 Area log (DAPI). Real time data analysis was displayed and interpreted using Summit flow system operating software v 6.3.1.

### GLuc Assay

GLuc substrate, native coelenterazine (CTZ), was reconstituted at 2.7 mg/mL in 200-proof ethanol. Working solutions were made fresh, immediately prior to assay with a 1:1000 dilution in PBS. Twenty microliters of sample or standard was added to black-walled, clear-bottom 96-well plates, and 100 μL of CTZ solution and immediately read using a bioluminescent plate reader (Varioskan, Thermo Fisher). All samples were read forward and in reverse to account for any time-dependent signal degradation. The concentration of GLuc in each sample was calculated by taking the average of the forward and reverse readings. Assays were performed under controlled lighting conditions with minimal exposure, and the temperature was maintained consistent throughout the experiments.

### Proliferation Assays

DS-1 cells were washed with PBS and kept in IL-6-free culture medium overnight. 30,000 cells were plated in 48-well plates in a final volume of 300 μl with reagents and/or inhibitors. Recombinant human IL-6 and human sIL-6R (R&D Systems) were pre-incubated for 30 min for complexation. Recombinant human sgp130 was also purchased from R&D Systems. Cells were cultured for 5 days and CellTiter-Blue Viability Reagent (Promega) was used to determine cellular viability by recording the fluorescence (excitation 579 nm, emission 584 nm) using Varioskan plate reader immediately after adding 20 μl of reagent per well (time point 0) and up to 240 min thereafter. Cell count and viability were also obtained via NucleoCounter® instrument (Chemometec).

### Transwell Co-culture Assays

DS-1 and Ba/F3 cells were washed with PBS and kept in IL-6-free culture medium overnight. 1 × 10^5^ engineered or control HEK cells were seeded in cell culture inserts for 6-well plates with 1 μm pore diameter (Thincert, Greiner Bio, ref # 657,610). Next day, 3 × 10^5^ DS-1 or Ba/F3 cells were seeded in 6-well plates with either IL-6 (50ng/mL) + sIL-6R (100ng/mL)—allowed to complex in advance for 30min at room temperature-, or Hyper IL-6 (10ng/mL), or none (untreated control group). Cell inserts containing adherent HEK cells were placed on top of each respective well. Cells were co-cultured for 8 days and CellTiter-Blue Reagent was used to determine cellular viability as previously described. Cell counts were obtained via NucleoCounter® instrument. Cell staining was performed at day 8 with Calcein AM (2 μM) and Hoechst (1 μg/mL) dyes and fluorescence was imaged and quantified using Celigo Imaging Cytometer (Nexcelom Bioscience).

### Animal Experiments

#### In Vivo Studies

NSG (NOD scid gamma) mice between 8 and 10 weeks old were used for all studies (Jackson Laboratories). Animals were housed at no more than three mice per cage and were allowed food and water ad libitum throughout the duration of the study. Within 1 week of arrival, mice were placed randomly into groups and identified using ear punches. For pharmacokinetic assessments, volumes of 500 μl and 300 μl were administered via i.p. and s.c. injections, respectively. For tumor xenograft model, 3 × 10^6^ DS-1 cells, 1 × 10^6^ hMSCs and 1 × 10^5^ engineered or control HEK cells were embedded in Matrigel™ Membrane Matrix (Corning, ref #354,248) at a 1:1 dilution with ice cold sterile PBS and injected s.c. in the dorsal flank of mice. Tumor measurements were performed using an external caliper. The greatest longitudinal diameter (length = L) and the greatest transverse diameter (width = W) were used to calculate tumor volumes (V_T_) with the formula V_T_ = 0.5 × L × W^2^. Blood draws were performed at the timepoints indicated, using a tail vein restrainer and heparinized blood tubes. Blood samples were immediately centrifuged at 2000 g for 5 min after each collection and plasma was carefully removed from cell pellet. When tumor volumes were ≥ 1800–2000 mm^3^ (humane endpoint criteria), animals were deeply anesthetized in isoflurane induction chamber until lack of response and cardiac puncture was performed for blood collection and tumor harvesting. Tissues were snap frozen in liquid nitrogen or fixed in 10% formalin for histology and immunohistochemistry (IHC) analyses. Spontaneous deaths were also recorded and included in analysis of survival curves. Blood was later analyzed for GLuc and sgp130Fc levels, as previously described. All animal work was performed in accordance with the ethical standards of the Institutional Animal Care and Use Committee (IACUC).

#### Histology and Immunohistochemistry

Histological analyses were performed at Rutgers Research Pathology Services Core Facility. Sample preparation included wet trimming, tissue cassetting, embedding in paraffin wax, and sectioning. Paraffin-embedded tissues were sectioned at 5 μm and stained with hematoxylin and eosin. For IHC, tissue sections were stained with anti-CD20 and anti-Ki67 antibodies, followed by anti-Histag secondary antibody. Photomicrography was conducted using a Leica microscope (2× objective). Images were processed and analyzed with Aperio Image Scope and ImageJ software [26].

#### Measurement of sgp130Fc Variant Levels

sgp130Fc and sgp130^FlyR^Fc levels were quantified in cell culture supernatants and blood plasma using a commercial human sgp130 ELISA kit (DuoSet kits from R&D Systems).

### Statistical Analyses

Experimental data were analyzed using GraphPad Prism 9.5.1 for Mac OS X (GraphPad Software, La Jolla, CA). The results are shown as the mean ± standard deviation (SD) and significance is set at *p* ≤ 0.05. Unpaired *t* test assuming equal variance between groups was used to compare ELISA data. For multiple comparisons, one-way or two-way analysis of variance (ANOVA) with Tukey’s multiple comparisons post-test were used unless otherwise noted. For survival studies, a Kaplan–Meier (log rank) test was used.

## Results

### DS-1 Cells as a Model for Anti-IL-6 Therapy Validation

DS-1 cells are an IL-6-dependent human B-cell lineage derived from a lymphoma immunodeficient patient, that has been employed in translational applications since it was first described by Bock et al. in 1993 [27]. Herein, we corroborate the use of DS-1 cells as an accessible tool for anti-IL-6 therapy characterization and validation. Notably, these cells express mIL-6R and can proliferate upon the activation of both IL-6 classic and/or trans-signaling. Considering that our focus was to evaluate the effect of trans-signaling inhibitors on cell proliferation, we took advantage of the intermolecular affinities between IL-6, sIL-6R, and sgp130 to skew the equilibrium toward trans-signaling. It is known that sIL-6R and sgp130 can act as systemic buffers when present in molar excess over IL-6, favoring the formation of IL-6/sIL-6R/sgp130 complexes, and consequent inhibition of IL-6 trans-signaling [28, 29]. However, at higher sgp130 concentrations, classic signaling can also be impacted due to lower availability of free, non-complexed IL-6.

**Fig. 1:**
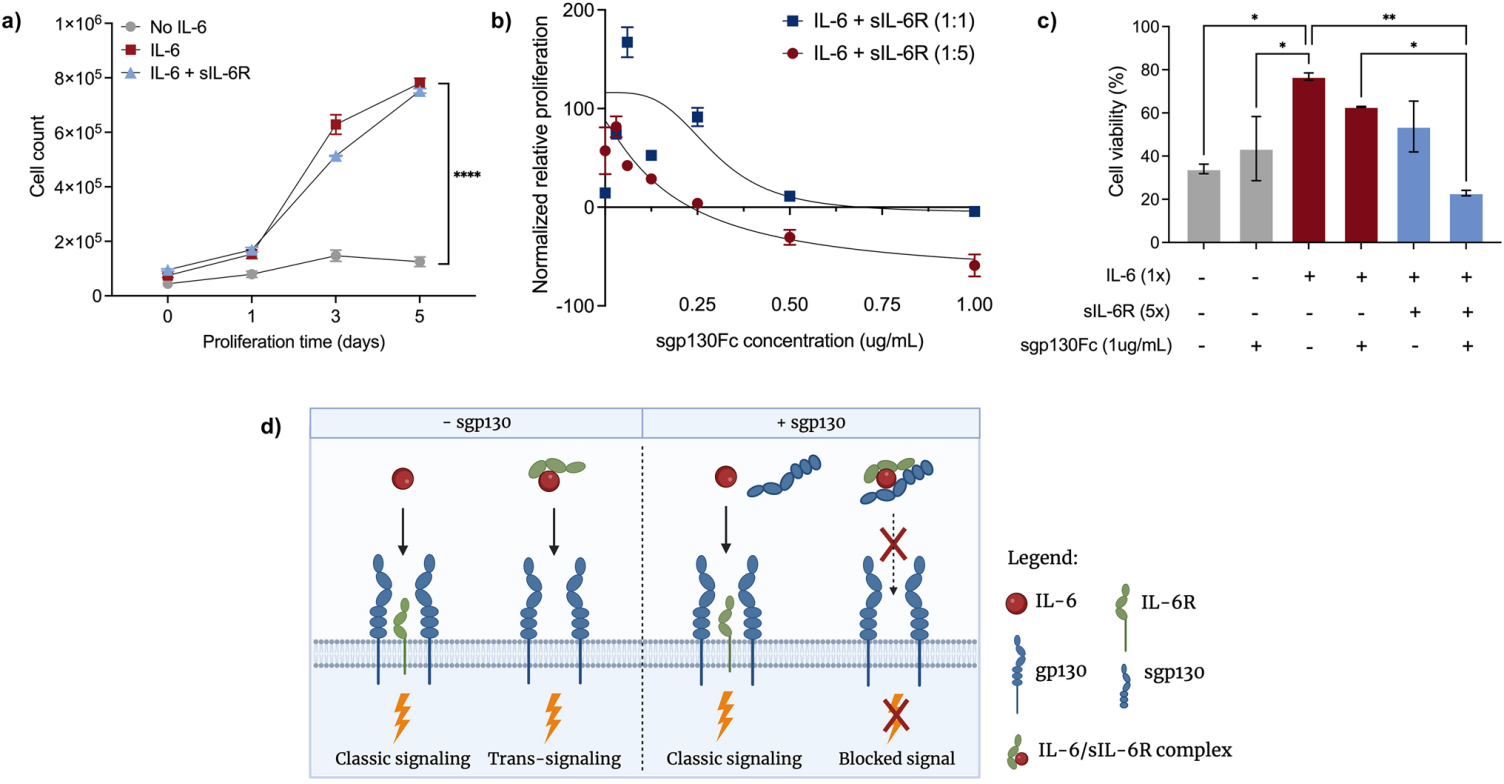
DS-1 cells can be used as a tool for anti-IL-6 therapy characterization **a** DS-1 cells were cultured in IL-6-free media, or in the presence of IL-6 and/or sIL-6R and cell count was obtained at the indicated timepoints (*n* = 3; means ± SD, ****p ≤ 0.0001 by AUC and unpaired t test) **b** DS-1 cells were stimulated with IL-6 + sIL-6R at different ratios in the presence of increasing concentrations of sgp130Fc. Five days after stimulation, cellular proliferation was detected via CellTiter-Blue assay. Relative proliferation was normalized to unstimulated control group (*n* = 3) **c** DS-1 cells were culture under different conditions, and cell viability was obtained at day 5 via automated cell counter (NucleoCounter®) (*n* = 3; means ± SD, *p ≤ 0.05, **p ≤ 0.01, by Two-way ANOVA and Tukey’s test) **d** Schematic of known mechanism through which endogenous sgp130 selectively inhibits IL-6 trans-signaling pathway, without interfering with IL-6 classic signaling (created with BioRender.com)

Consistently, when cultured in the presence of IL-6 or IL-6 + IL-6R, DS-1 cells proliferated exponentially (Fig. 1a). Conversely, when IL-6 pathway was inhibited by the presence of first-generation sgp130Fc, cell proliferation was impaired in a dose-dependent manner (Fig. 1b). Particularly, sgp130Fc exhibited a near-linear inhibitory effect when IL-6 concentration was lower than that of sIL-6R (1:5 ratio). The same was not observed when there was no molar excess of sIL-6R over IL-6, especially in the presence of lower doses of sgp130, corroborating the low affinities and dissociation constant (K_d_) values for IL-6 and IL-6R in the absence of sgp130 [28, 30]. Therefore, in that scenario, IL-6 is not complexed to sIL-6R and is free to engage mIL-6R and activate the classic signaling downstream, resulting in cell proliferation. Similarly, cell viability was impaired in the absence of IL-6 and by the recombinant inhibitor only in the presence of IL-6 + sIL-6R (Fig. 1c), suggesting its inhibitory effect on IL-6 trans-signaling pathway. Figure 1d illustrates the known mechanism through which soluble gp130 binds to the IL-6/sIL-6R complex, preventing it from engaging with the membrane-bound gp130 receptor, and therefore, inhibiting the trans-signaling pathway activation.

### Effective In Vitro IL-6 Inhibition via Recombinant Engineered Therapeutics

**Fig. 2:**
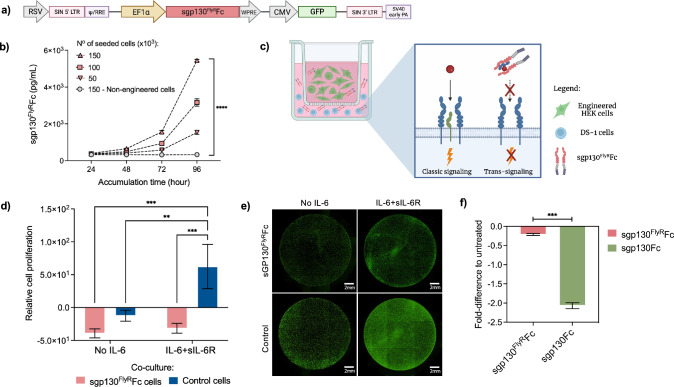
Second-generation engineered therapeutic exhibits great efficiency in inhibiting IL-6-dependent cell proliferation **a** Schematic of lentiviral genetic construct used to transduce target cells, encoding second-generation IL-6 trans-signaling inhibitor sgp130^FlyR^Fc **b** Engineered HEK cells were cultured for 96 h, and conditioned media were collected at indicated timepoints and assessed for recombinant protein secretion (*n* = 3; means ± SD, ****p ≤ 0.0001 by AUC and unpaired *t* test) **c** Illustration of co-culture assay performed using trans-well insert to assess bioactivity of secreted therapeutics as a function of DS-1 cell proliferation. Colored shapes have the same meanings as in Fig. 1 (created with BioRender.com) **d** DS-1 cells were co-cultured with control or sgp130.^FlyR^Fc-secreting HEK cells, in the absence or presence of the IL-6 + sIL-6R. 8 days after stimulation, cellular proliferation was detected using a CellTiter-Blue assay. Results show cell proliferation relative to day 0 (*n* = 3; means ± SD, **p ≤ 0.01; ***p ≤ 0.001, two-way ANOVA) **e** Calcein cellular staining was performed at day 8 and images were obtained using imaging cytometer **f** DS-1 cells were co-cultured with sgp130Fc-secreting HEK cells under same parameters as described in Fig. 2D. Comparative analysis was performed in relation to untreated control groups for both first and second-generation inhibitors. Data shown are means ± SD, ***p ≤ 0.001, unpaired *t* test (*n* = 3)

We sought to explore the high protein producing capacity of HEK293T cells by using them as vehicles of engineered anti-IL-6 therapeutics. Cells were stably transduced with lentiviral vectors encoding the second-generation IL-6 trans-signaling inhibitor sgp130^FlyR^Fc under the control of the constitutive human elongation factor-1 alpha (EF1α) promoter (Fig. 2a). A green fluorescent protein (GFP) marker was also included for transduction efficiency determination and cell sorting purposes. Sorted engineered cells yielded increasing amounts of the inhibitory protein, detected over time in the supernatant in a cell-density-dependent manner (Fig. 2b). The calculated transgene secretion rate was approximately 55pg/10^6^ cells/day (**Online Resource 1a**). To characterize the bioactivity of secreted therapeutics, DS-1 cells were co-cultured with either engineered sgp130^FlyR^Fc-secreting HEK cells or control HEK cells using a trans-well insert, as depicted in Fig. 2c. Assuming a continuous secretion of engineered inhibitor through the trans-well, proliferation of DS-1 cells was significantly impaired by the presence of sgp130^FlyR^Fc in the supernatant. Conversely, in the presence of control HEK cells and IL-6 + sIL-6R, DS-1 cell proliferation remained unaffected (Fig. 2d). Viability staining at the end of study revealed significantly lower number of Calcein^+^ cells (**Online Resource 1b**) in groups co-cultured with engineered therapeutic cells, confirming reduced viability (Fig. 2e).

To establish the efficacy of second-generation secreted therapeutics, a comparative study was performed using the first-generation IL-6 trans-signaling inhibitor sgp130Fc. HEK293T cells were stably transduced using lentiviral vectors and sorted via flow cytometry. Inhibitory protein was detected in the supernatant over time and secretion dynamics were monitored. Notably, the calculated secretion rate was very similar to that observed for second-generation inhibitor (**Online Resource 1c**), revealing comparable protein concentrations over time. Engineered cells were then co-cultured with DS-1 cells, in the presence or absence of IL-6 + sIL-6R, of as previously described.

DS-1 cell proliferation was compared to that of untreated group (cultured in the absence of IL-6 + sIL-6R) and a fold-difference was calculated for each inhibitor (**Online Resource 1d**). The second-generation inhibitor sgp130^FlyR^Fc demonstrated approximately tenfold greater efficacy in replicating the effects observed in the untreated group, indicating a greater capacity for IL-6 inhibition. (Fig. 2f).

### Selective Inhibition of IL-6-*trans*-Signaling Pathway

**Fig. 3:**
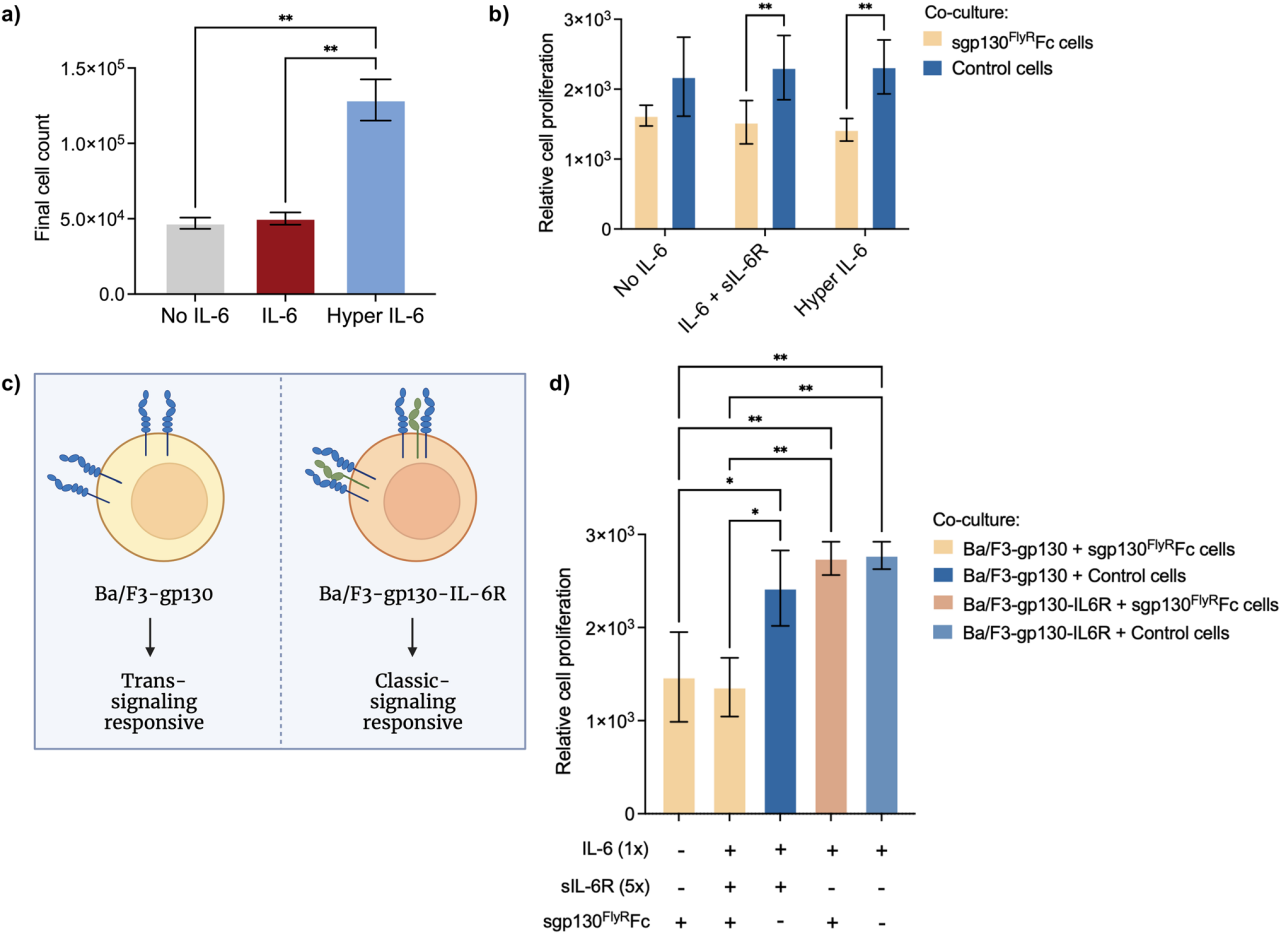
Secreted therapeutic is selective toward IL-6 trans-signaling pathway **a** Ba/F3-gp130 cells were cultured in IL-6-free media or in the presence of IL-6 or Hyper IL-6 (10ng/mL) and cell count was obtained after 5 days of expansion (*n* = 3) **b** Ba/F3-gp130 cells were co-cultured with control or sgp130^FlyR^Fc-secreting HEK cells, in the presence or absence of IL-6 + sIL-6R (1:5 ratio) or Hyper IL-6. 8 days after stimulation, cellular proliferation was detected using a CellTiter-Blue assay (*n* = 4). Results show cell proliferation relative to day 0 **c** Illustration of stably transduced Ba/F3 cell lines, that can exhibit either selective trans-signaling responsiveness or become sensitive to classic signaling upon further transduction with mIL-6R. Colored shapes have the same meanings as in Fig. 1 (created with BioRender.com) **d** Ba/F3-gp130-IL-6R cells were co-cultured with control or sgp130.^FlyR^Fc-secreting HEK cells in the presence of IL-6. Cellular proliferation was obtained at day 8 and compared to results obtained for Ba/F3-gp130 cells under similar conditions (*n* = 3; means ± SD; *p ≤ 0.05, **p ≤ 0.01, two-way ANOVA)

Next, we sought to confirm the selectivity of secreted inhibitor toward IL-6 trans-signaling by using Ba/F3 cells as a model system. Ba/F3 are a murine pro-B-cell line that proliferates in response to IL-6-trans-signaling upon stable transduction with gp130 (Ba/F3-gp130) [17, 31]. First, we stimulated Ba/F3-gp130 cells with either IL-6 or Hyper-IL-6 (fusion protein of IL-6 and sIL-6R). A significantly higher cell proliferation was observed in the presence of Hyper IL-6, compared to groups that received IL-6 alone or no stimulation, confirming the exclusive responsiveness of these cells to trans-signaling pathway (Fig. 3a). Next, we co-cultured Ba/F3-gp130 cells with either sgp130^FlyR^Fc-secreting HEK cells or control HEK cells, in a similar manner as previously described. As expected, the proliferation of cells in control groups was significantly higher than the ones exposed to secreted anti-IL-6 therapeutics, in the presence of IL-6 + sIL-6R or Hyper IL-6 (Fig. 3b). To validate the specificity of secreted inhibitor toward IL-6 trans-signaling, we co-cultured sgp130^FlyR^Fc-secreting cells with Ba/F3 cells that are additionally stably transduced to express mIL-6R (Ba/F3-gp130-IL-6R) (Fig. 3c). By expressing the membrane-bound IL-6 receptor, these cells can now respond to IL-6 classic signaling in the presence of IL-6. Ba/F3-gp130-IL-6R cells exposed to secreted sgp130^FlyR^Fc and IL-6 proliferated significantly more than trans-signaling-responsive Ba/F3-gp130 cells, excluding an effect on classic signaling. Furthermore, Ba/F3-gp130-IL-6R cells proliferated at similar levels when co-cultured with control cells, when neither classic nor trans-signaling pathways were being inhibited (Fig. 3d).

### Pharmacokinetics of Engineered Construct*:* Gene Transfer vs. Cell-Based Delivery

**Fig. 4:**
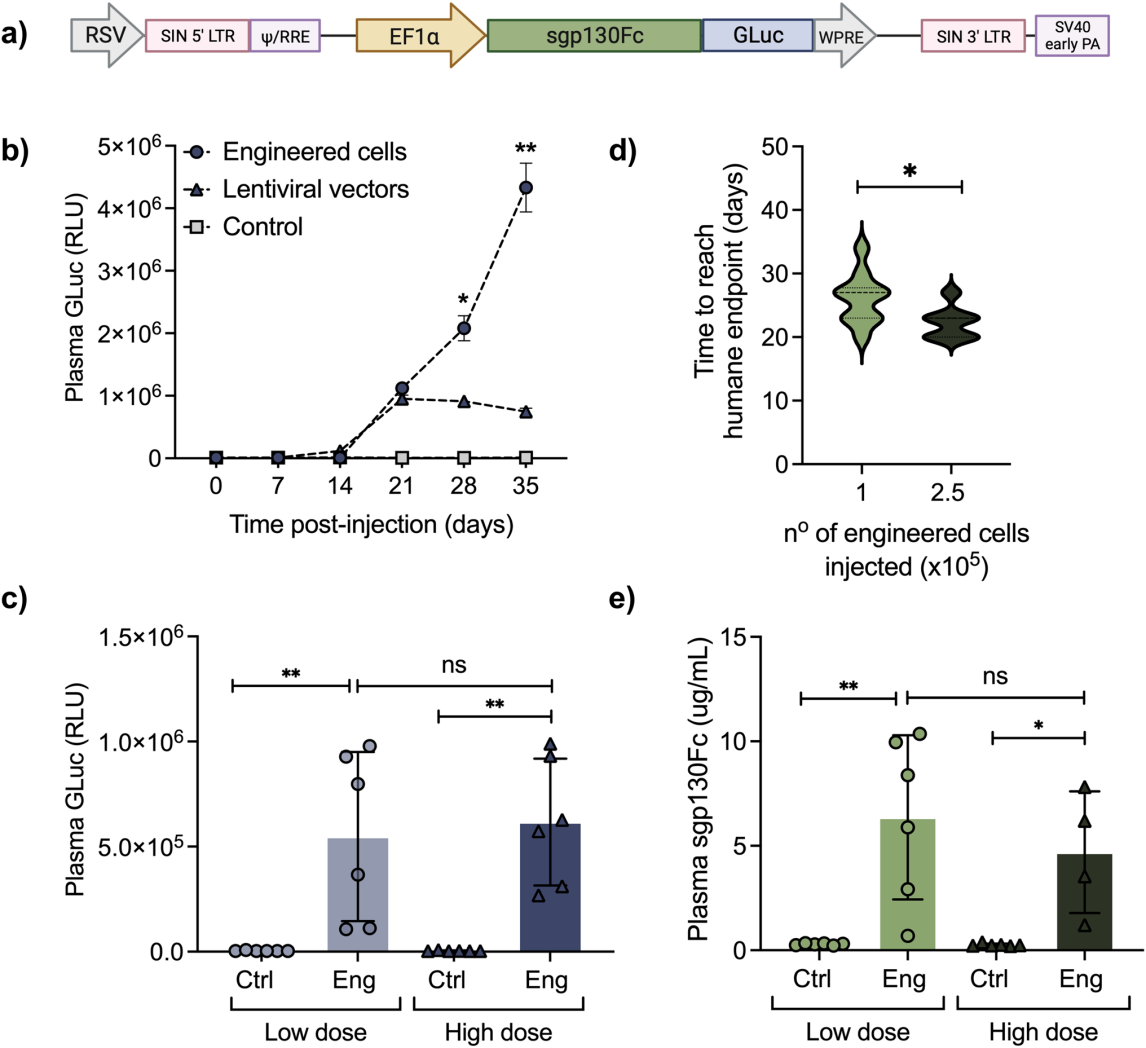
Cell-based delivery of recombinant anti-IL-6 therapeutics yields superior bioavailability **a** Schematic of lentiviral genetic construct encoding first-generation IL-6 trans-signaling inhibitor and secreted biomarker GLuc **b** NSG mice were injected with lentiviral vectors intraperitoneally, or with control or engineered HEK cells in a Matrigel scaffold subcutaneously (dorsal flank). Blood samples were collected at indicated timepoints and plasma was assessed for GLuc concentration **c** Mice received s.c. injections with either 1 × 10^5^ or 2.5 × 10^5^ engineered HEK cells. Control groups received same doses of non-engineered cells. Blood samples were collected when cell aggregates reached endpoint criteria and animals were sacrificed. Plasma was assessed for GLuc concentration **d** Time to reach humane endpoint criteria was recorded and compared between groups **e** Plasma levels of sgp130Fc at end of study were determined via ELISA. All data are from *n* = 6 animals/group, shown as means (horizontal bars) ± SD (vertical bars) and single values (symbols) or violin plot, *p ≤ 0.05; **p ≤ 0.01, one-way ANOVA or unpaired *t* test

To establish the bioavailability and pharmacokinetic parameters of secreted therapeutics in an in vivo mammalian system, a new genetic construct was designed encoding a bioluminescent biomarker probe—*Gaussia Luciferase* (GLuc) [32]—under the control of the constitutive promoter EF1α (Fig. 4a). This biomarker enables a quick and sensitive verification of protein level with just a few μl of blood, being suitable for initial in vivo parameter-finding steps. The first-generation inhibitor sgp130Fc was encoded under the same promoter, and selected for these screening studies because more information is currently available regarding its properties. Importantly, GLuc concentrations can be directly correlated with the inhibitor plasma concentration since both genes are being expressed *in tandem*. The construct was initially administered to mice in two formats: via gene transfer—using lentiviruses as delivery vectors; and as engineered cell products—using genetically modified HEK cells as vehicles of secreted therapeutics. Mice were injected with either 2.5 × 10^8^ infection units (IU)/kg intraperitoneally (i.p.) or engrafted with 5 × 10^5^ engineered HEK cells in Matrigel scaffold subcutaneously (s.c.). The viral dose was selected based on previous in vivo lentiviral studies [33]. The cell number was calculated based on in vitro findings, that enabled a guided dose selection considering the calculated protein secretion rate and a doubling time of 48h for HEK cells. Control group received the same number of non-engineered cells. Evidently, protein plasma concentration is dependent upon several other factors, such as protein half-life and clearance, so we recovered GLuc via blood sampling and monitored its secretion dynamics over time to elucidate in vivo secretory profile of both groups more precisely. Notably, cell-based delivery gave rise to the highest systemic levels of the biomarker, exhibiting exponential GLuc plasma detectability, whereas LV-injected group reached a plateau around day 21 post-injection (Fig. 4b). Therefore, cell-based delivery of therapeutics was adopted in the following studies.

To further characterize the pharmacokinetics of the construct—and because HEK cells exhibited a very quick expansion—we next tested other cell therapy doses aiming at a slower cell proliferation for better monitoring over time. Mice received either 1 × 10^5^ (low dose) or 2.5 × 10^5^ (high dose) engineered cells s.c. and GLuc was recovered via blood sampling at the end of study, when cell aggregates reached endpoint criteria and animals were sacrificed. Interestingly, there was no significant difference in GLuc plasma levels between both groups (Fig. 4c), yet the group that received the lowest cell dose exhibited a longer time to reach humane endpoint *(see Materials and Methods)* (Fig. 4d). Hence, the lower dose was adopted in the subsequent in vivo cell-based applications. Finally, to confirm the accuracy of results obtained based on biomarker detection, plasma levels of the IL-6 inhibitor were also assessed via ELISA. As expected, sgp130Fc systemic levels recapitulated results obtained with GLuc, with no significant differences between low and high dose groups at the end of study. Recombinant protein plasma concentration was around 7 μg/mL at day 35, which was within the expected range (Fig. 4e).

### In Vivo Bioactivity of Recombinant Therapeutics: Intratumoral Cell-Based Delivery in a Xenograft Lymphoma Model

**Fig. 5:**
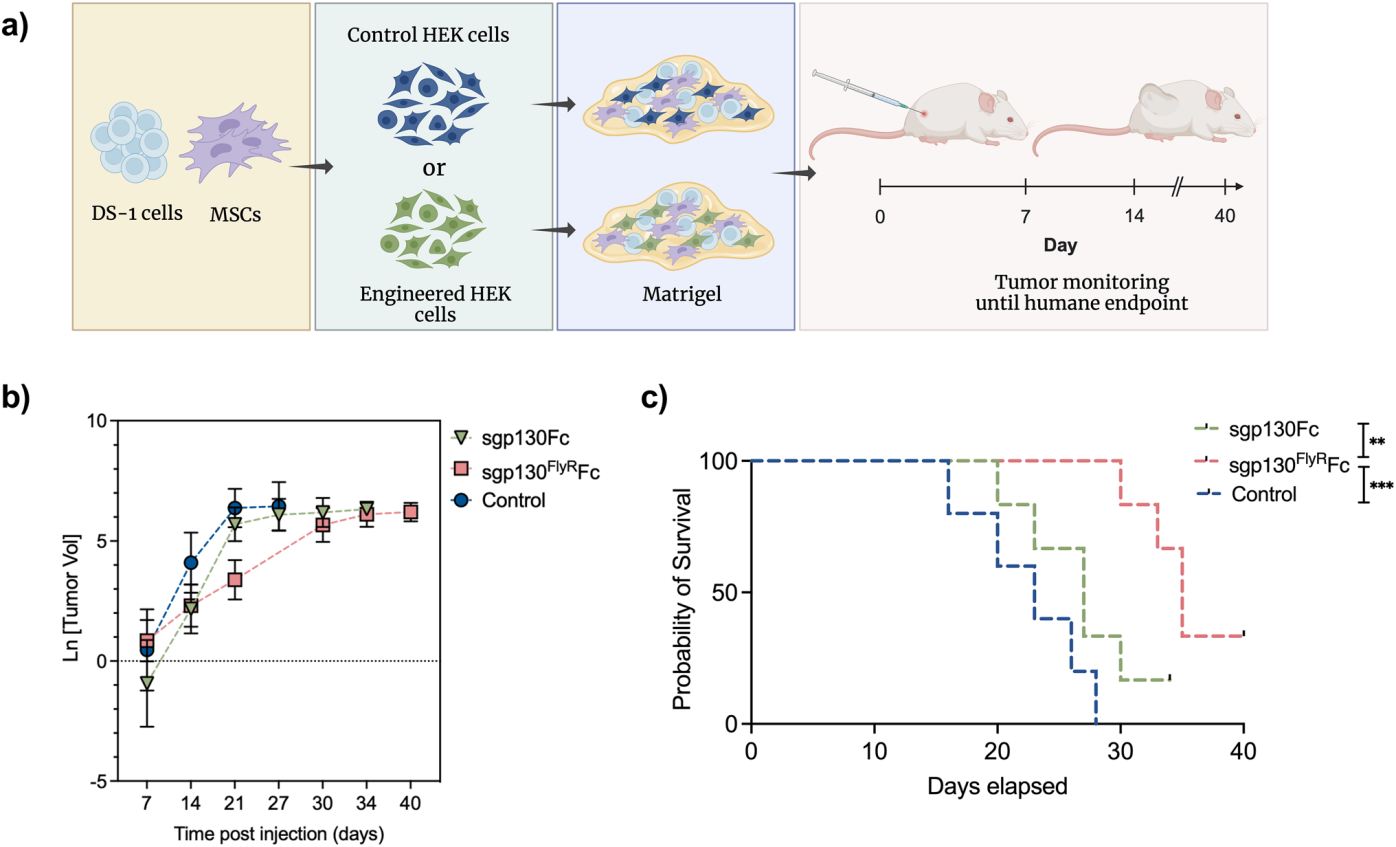
Intratumoral delivery of recombinant anti-IL-6 therapeutics is efficient in inhibiting IL-6-dependent tumor xenograft growth **a** Schematic of xenograft lymphoma model used to assess in vivo inhibitory capacity of recombinant anti-IL-6 therapeutics (created with BioRender.com) **b** Tumor growth progression post-xenograft injection. Tumor volumes were monitored and recorded at indicated timepoints; animals were monitored until reaching humane endpoint **c** Survival curve comparing first- and second-generation inhibitors and controls. All data are from *n* = 6 animals/group, shown as means ± SD. Survival analysis was generated via Kaplan–Meier estimate and Mantel-Cox test (**p ≤ 0.01; ***p ≤ 0.001)

Recombinant protein production and purification are expensive and time-consuming processes, that pose challenges to the use of anti-cytokine therapies in clinic. Herein, we sought to establish a proof-of-concept framework that enables local cell-based delivery of anti-cytokine therapeutics, yielding high levels of soluble protein with mensurable inhibitory effects. To determine the in vivo bioactivity and inhibitory potency of recombinant therapeutics, a xenograft lymphoma model was developed (Fig. 5a). Using a Matrigel scaffold, IL-6-dependent DS-1 cells served as the tumor xenograft model, and human mesenchymal stem cells (hMSCs) served as a stromal IL-6 feeding-layer, based on their secretory profile [34–36]. Engineered anti-IL-6-secreting cells were added to the scaffold for intratumoral delivery of anti-cytokine therapeutics. Control groups received non-engineered HEK cells. Xenografts were engrafted into immunodeficient NSG mice, to circumvent rejection of human cells, and tumor proliferation was monitored over time, until reaching humane endpoint *(see Materials and Methods)*. An initial exploratory study investigated first-generation sgp130Fc-secreting cells’ inhibitory capacity as compared to controls. Tumor volumes were slightly lower in the treated group (**Online Resource 2a**) and, most importantly, survival of treated animals was somewhat higher than controls (**Online Resource 2b**), suggesting IL-6 inhibition and impairment of DS-1 cell proliferation. Histology analysis (H&E) did not show major differences between groups (**Online Resource 3a**). IHC was performed for CD20—a general B-cell marker-, and Ki67—a marker of cell growth and proliferation. Control group exhibited slightly higher number of double positive cells (CD20^+^Ki67^+^), indicating a prevalence of proliferative lymphoma cells (**Online Resource 3b**).

Finally, second-generation anti-IL-6-secreting cells were tested using the same xenograft model, and their inhibitory efficiency was compared. Tumor progression exhibited a different pattern in each group, with a slower and more sustained tumor growth rate in the sgp130^FlyR^Fc treated group, as shown in Fig. 5b. Importantly, survival of animals engrafted with sgp130^FlyR^Fc-secreting cells was significantly higher than controls or first-generation therapeutics (Fig. 5c). These results underscore the enhanced efficacy of the next-generation inhibitor, validating the previously observed outcomes in vitro. Altogether, these studies bring a new perspective and lay a foundation for sustained delivery of anti-cytokine therapeutics via genetically engineered modalities.

## Discussion

The knowledge on IL-6 biology has expanded considerably over the last decades. Pan-inhibitors of IL-6 signaling were first developed and approved in several countries for the treatment of chronic inflammatory diseases, such as rheumatoid arthritis, Crohn’s disease, and Castleman disease [14]. Later, when IL-6 trans-signaling was proven to be the pathological arm of IL-6, selective inhibitors emerged and have been showing efficacy in multiple clinical and pre-clinical models of inflammation and cancer. In fact, treatment with recombinant sgp130Fc has shown superior results over global IL-6 signaling blockade in several disease models, such as myocardial infarction, sepsis, and acute pancreatitis [5, 37, 38]. Next-generation inhibitors have taken anti-IL-6 trans-signaling to the next level, exhibiting higher affinity, specificity, and bioavailability [5]. Despite that, anti-cytokine therapy has faced setbacks due to the intricate and expensive nature of processes involved in recombinant protein and monoclonal antibody production and purification. Besides, during the early phases of development, factors such as shelf-life, storage requirements, and global distribution logistics are often overlooked [39]. Herein, we validated the superior inhibitory potency of second-generation sgp130^FlyR^Fc using an IL-6-dependent lymphoma cell line and xenograft tumor model and established a proof-of-concept gene therapeutic approach for sustained intratumoral delivery of anti-cytokine therapeutics, that can serve as an alternative for scaled-up manufacturing and repeated systemic administration.

IL-6-dependent DS-1 cell line enabled the validation and pharmacokinetic characterization of engineered anti-IL-6 trans-signaling therapeutics, both in in vitro and in vivo settings. Importantly, cells proliferated in the presence of both IL-6 and IL-6 + sIL-6R, confirming their responsiveness to both classic and trans-signaling IL-6 pathways. Considering that mIL-6R is only expressed on the surface of few cell types, such as hepatocytes, epithelial cells, and lymphocytes [1], DS-1 cells are naturally responsive to both IL-6 signaling pathways, as previously mentioned. This makes this cell line a reliable model to assess global IL-6 inhibition potential of drug candidates, but not to prove the specificity of a given drug toward trans-signaling pathway. To dissect each of these pathways and attest the specificity of secreted therapeutics, we used the well-established Ba/F3 cell model system. In fact, proliferation of only IL-6 trans-signaling-responsive cells was hindered by the presence of trans-signaling inhibitor, not affecting classic-signaling-responsive cells. Both cell models in combination can be a useful tool to dissect IL-6 signaling pathways, synergistically. For a more comprehensive and in-depth mechanistic characterization, however, the underlying molecular basis and intracellular mechanisms could be dissected. Because these have been extensively described for IL-6 pathways, our efforts were mostly focused on the relevant readouts for our proposed model, i.e., cell proliferation, pharmacokinetic parameters, and tumor burden/survival.

In vivo, sustained intratumoral secretion of the trans-signaling inhibitor reduced tumor burden. We were able to demonstrate increased animal survival and diminished lymph node expansion. Importantly, while the hMSC feeding-layer primarily served as an IL-6 source, it is crucial to acknowledge the presence of sIL-6R within the tumor microenvironment. This is supported by the physiological generation of mIL-6R through proteolysis or alternative splicing, with subsequent shedding by cells into the interstitium, as previously outlined. Also, since stromal elements were supporting DS-1 cell proliferation, these could also have been targeted by IL-6 trans-signaling inhibitor, leading to diminished local IL-6 secretion and overall impairment of stromal and lymphoma cell proliferation. Control groups served as a reference for the potential impact of basal MSC secretion or HEK cell proliferation, which showed qualitative expansion of these stromal cell types on tumor burden and animal survival. Exploratory study with first-generation inhibitor showed some histopathological changes that are in line with a direct impact of recombinant therapeutics on DS-1 cell proliferation; however, further mechanistic investigation would be needed for more conclusive evidence. Nevertheless, a clear therapeutic effect was demonstrated, supporting the potential of this approach. Future avenues could leverage the application, by exploring the combination of anti-cytokine therapeutics and cancer killing agents for synergistic anti-tumor effects in cytokine-dependent tumor models.

Gene and cell-based therapies have emerged as potential alternatives for sustained and controlled protein production, bypassing the challenges associated with poor bioavailability and biodistribution, low specificity, intricate biomanufacturing processes, as well as issues concerning patient compliance and tolerability to long-term treatments [22, 24, 40, 41]. Currently, there are multiple engineered cytokine therapeutics undergoing clinical trials, including plasmid and mRNA therapeutics for optimized cytokine expression, mainly targeting auto-immune diseases and metastatic solid tumors, as well as cell therapies, such as IL-12 expressing TCR-T cells and IL-15 expressing CAR NK cells for refractory carcinomas and lymphoid tumors [39]. This underscores the significance of our study in exploring analogous modalities for delivery of anti-IL-6 gene therapeutics. It is important to note that the ultimate plasma concentration of a given transgene is highly complex and depends on several parameters, such as volume of distribution and elimination rate, as well as the overall physiology of the animal [24]. Direct gene delivery via viral or non-viral vectors further relies on parameters such as initial distribution throughout the body, binding to cells, translocation, and unpackaging for optimal gene expression [24]. Intratumoral delivery can circumvent some of these challenges and enable sustained local secretion of transgene over time, minimizing off-target effects. Previous studies have shown that mRNA therapies encoding a cytokine cocktail were able to reshape the tumor microenvironment when injected intratumorally [39, 42, 43]. By initially establishing the secretion rate of engineered therapeutics in vitro and analyzing their inhibitory potential relative to the number of target tumor cells, we were able to perform a guided dose selection for in vivo applications. Our proof-of-concept study, demonstrating intratumoral delivery of engineered therapeutics, unveiled promising anti-cytokine effects that could be leveraged to clinically relevant applications. Local delivery of therapeutic cells may not be a viable option for some disease types; however, target cells such as tumor infiltrating lymphocytes, macrophages, dendritic cells or NK cells, can be genetically modified for enhanced tissue localization when delivered systemically. In fact, studies have shown that genetically engineered myeloid cells expressing IL-12 were able to reduce tumor burden and improve survival in metastatic tumor models, with minimal off-target effects [44]. IL-15 transpresenting dendritic cells were also able to activate tumor-targeting NK cells, reducing the growth of acute myeloid leukemia cells [45]. Finally, genetically modified oncolytic viruses also represent an interesting avenue for targeted delivery of anti-IL-6 trans-signaling therapeutics [46]. In that case, however, the vectors’ payload capacity might represent a limitation, depending on the size of the transgene of interest.

In summary, our results confirm the superior inhibitory potential of second-generation anti-IL-6 trans-signaling and lay a foundation for anti-cytokine delivery as a gene therapeutic modality. Cell-based delivery of engineered pharmaceuticals can be a viable solution to bypass the time-consuming and costly production and purification of monoclonal antibodies, while providing a self-sustaining solution to drugs that require repeated administration regimens.

## Supplementary Information

Below is the link to the electronic supplementary material.

Supplementary file1 (TIFF 18214 KB)

Supplementary file2 (TIFF 18214 KB)

Supplementary file3 (TIFF 20764 KB)

Supplementary file4 (PDF 603 KB)

Supplementary file5 (PDF 325 KB)

Supplementary file6 (PDF 2264 KB)

## Data Availability

The original contributions presented in the study are included in the article/supplementary material, further inquiries can be directed to the corresponding author.
