# Supplementary figures and images for: Intratumoral Delivery of Genetically Engineered Anti-IL-6 Trans-signaling Therapeutics
Source: Mol Biotechnol. 2024 Jul 9;67(7):2696–708. doi: 10.1007/s12033-024-01230-6 (PMC12119671; doi:10.1007/s12033-024-01230-6)

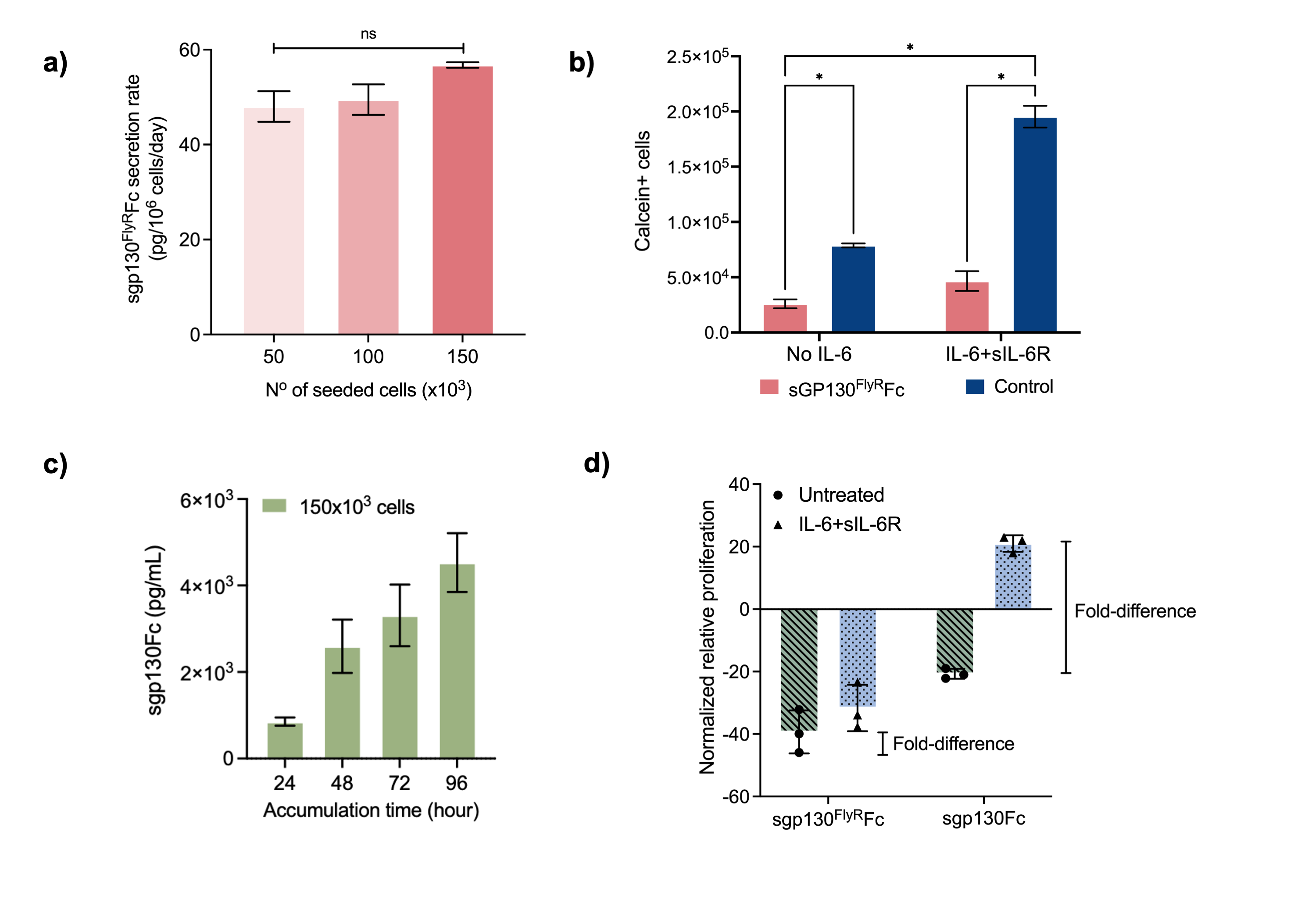

Supplement: Supplementary file 1 — Supplementary file1 (TIFF 18214 KB) [file 12033_2024_1230_MOESM1_ESM.tiff]

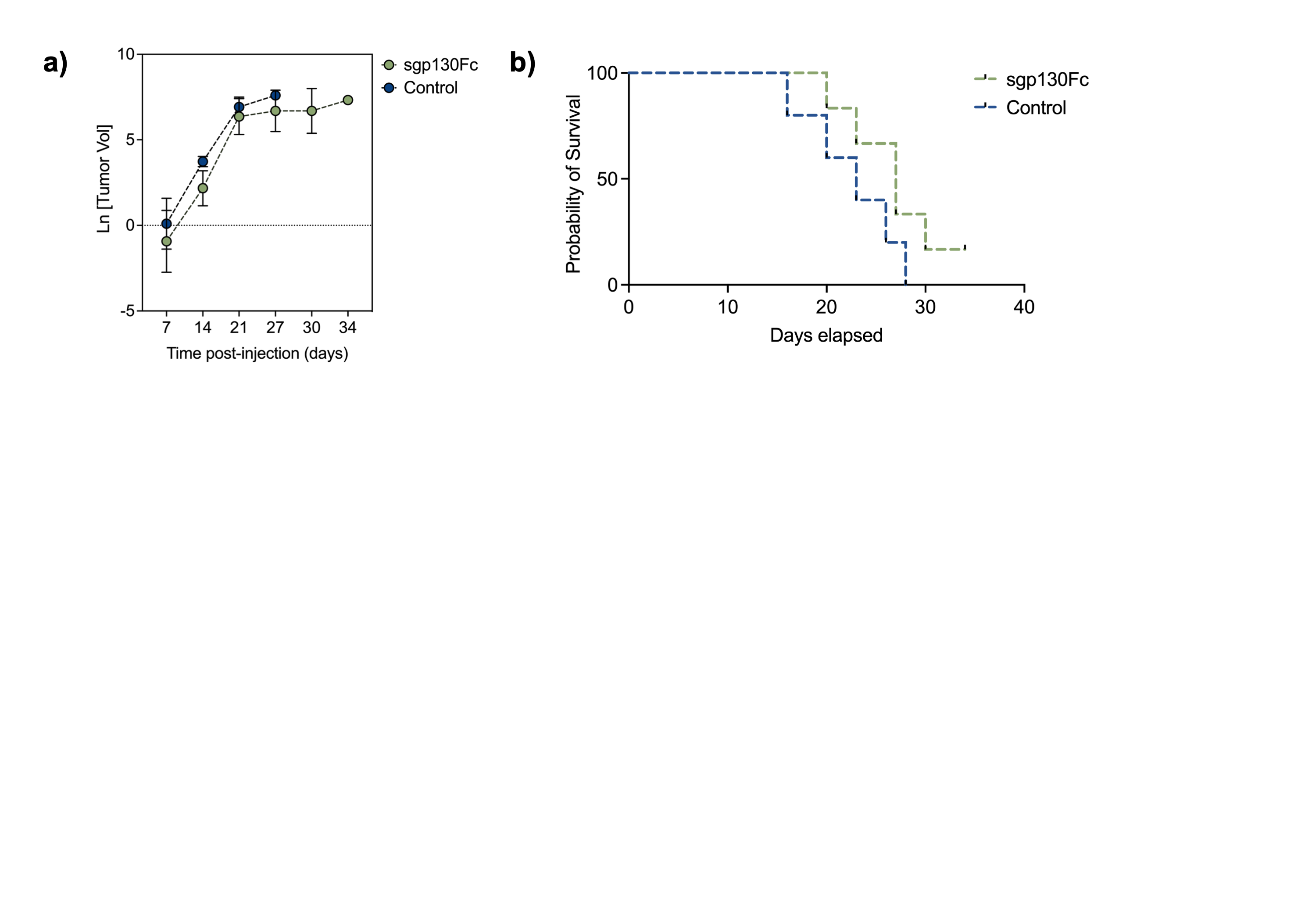

Supplement: Supplementary file 2 — Supplementary file2 (TIFF 18214 KB) [file 12033_2024_1230_MOESM2_ESM.tiff]

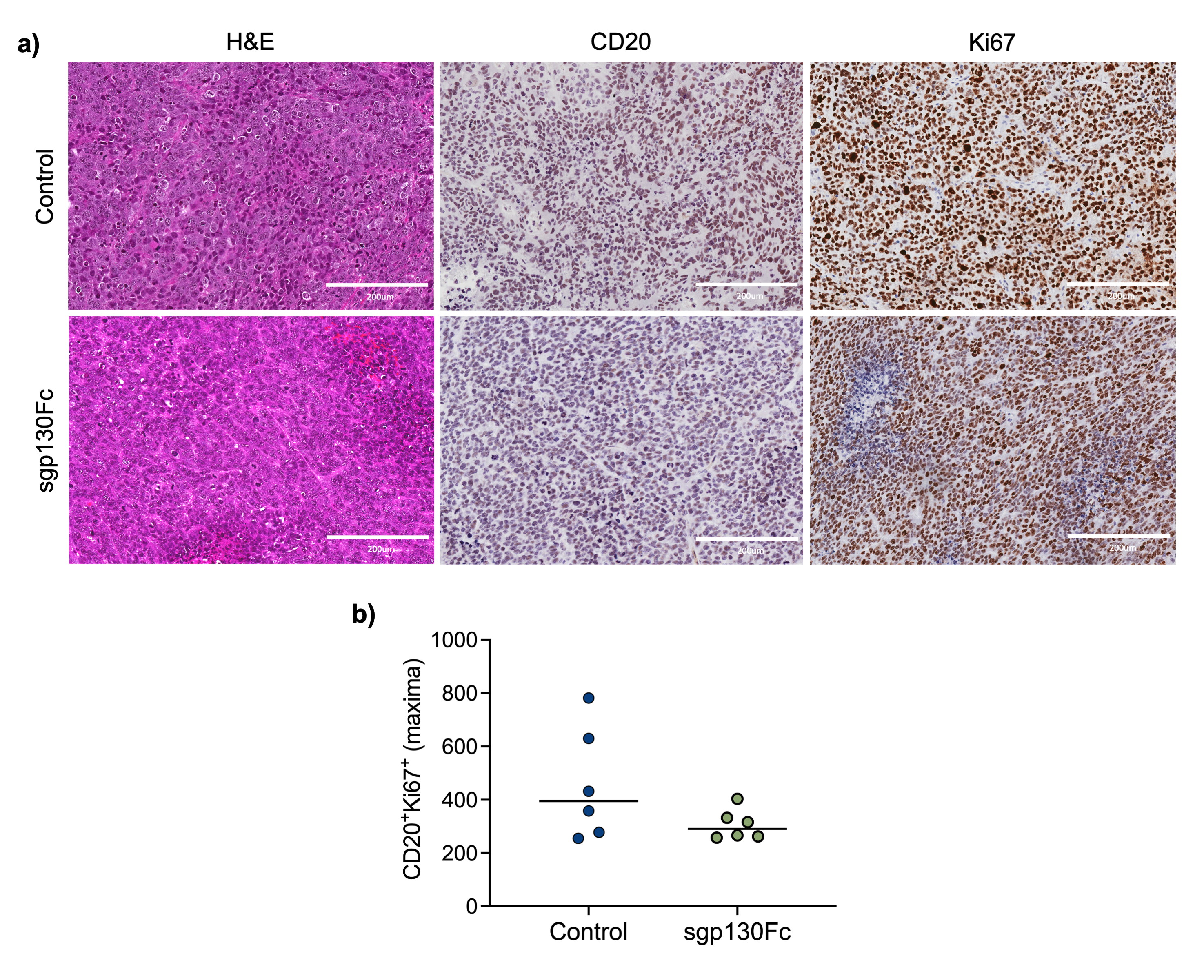

Supplement: Supplementary file 3 — Supplementary file3 (TIFF 20764 KB) [file 12033_2024_1230_MOESM3_ESM.tiff]
