# Supplementary material for: Intratumoral Delivery of Genetically Engineered Anti-IL-6 Trans-signaling Therapeutics
Source: Mol Biotechnol. 2024 Jul 9;67(7):2696–708. doi: 10.1007/s12033-024-01230-6 (PMC12119671; doi:10.1007/s12033-024-01230-6)
Supplement: Supplementary file 4 — Supplementary file4 (PDF 603 KB) [file 12033_2024_1230_MOESM4_ESM.pdf]

# Intratumoral delivery of genetically engineered anti-IL-6 trans-signaling therapeutics

## Online Resource 1

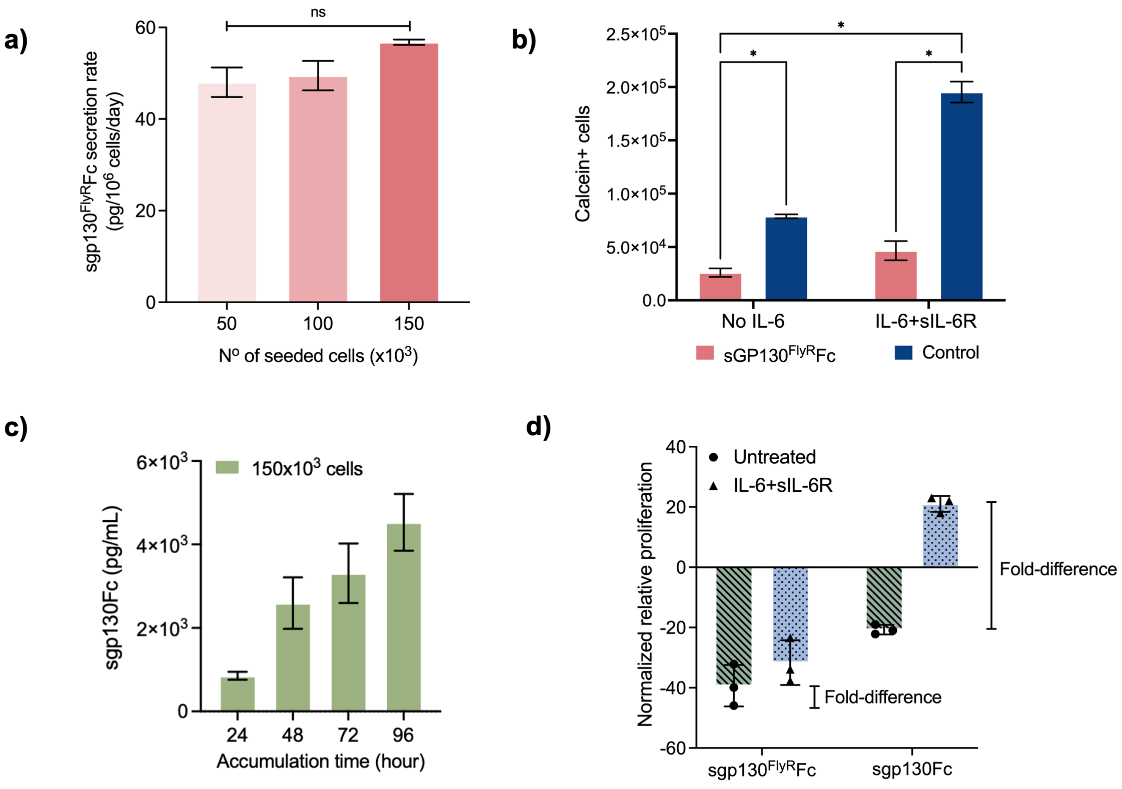


**Online Resource 1 a)** Secretion rate of engineered HEK cells relative to initial cell seeding number. Recombinant protein secretion was consistent throughout groups, with an average secretion of about 55pg/$10^6$ cells/day **b)** Quantification of Calcein$^+$ DS-1 cells, confirming reduced viability in groups treated with sgp130$^{FlyR}$Fc-secreting cells **c)** Secretion dynamics of first-generation sgp130Fc-secreting cells, with a seeding density of 150x$10^3$ cells **d)** Comparison of relative cell proliferation between groups co-cultured with first and second-generation trans-signaling inhibitors. Fold-difference to untreated groups was higher for first-generation inhibitor, suggesting reduced inhibitory potency
