# Supplementary material for: Intratumoral Delivery of Genetically Engineered Anti-IL-6 Trans-signaling Therapeutics
Source: Mol Biotechnol. 2024 Jul 9;67(7):2696–708. doi: 10.1007/s12033-024-01230-6 (PMC12119671; doi:10.1007/s12033-024-01230-6)
Supplement: Supplementary file 5 — Supplementary file5 (PDF 325 KB) [file 12033_2024_1230_MOESM5_ESM.pdf]

Intratumoral delivery of genetically engineered anti-IL-6 trans-signaling therapeutics

Online Resource 2

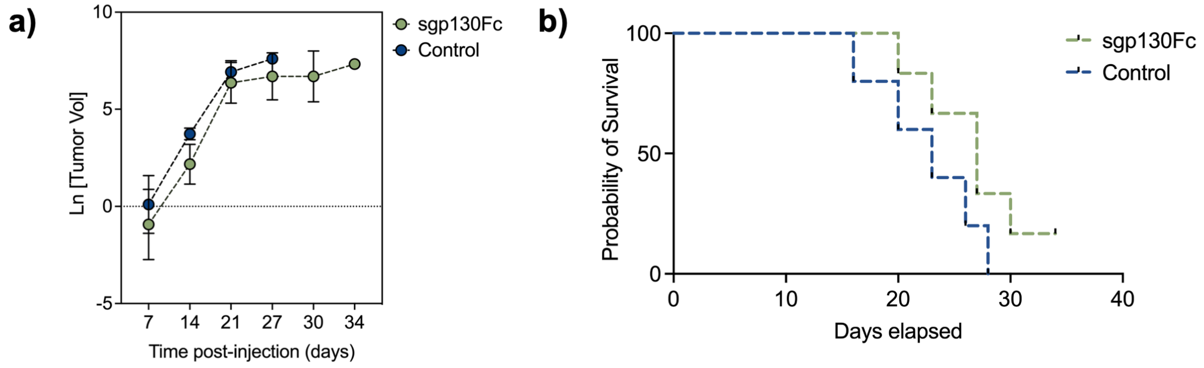

Online Resource 2 a) Tumor progression in groups treated with either sgp130Fc-secreting HEK cells or control HEK cells. Tumor volumes were monitored and recorded at indicated time-points **b)** Survival curves were generated based on time to reach humane endpoint criteria. Curves show slightly higher animal survival in treated group (n=6 per group)
