# Supplementary material for: Intratumoral Delivery of Genetically Engineered Anti-IL-6 Trans-signaling Therapeutics
Source: Mol Biotechnol. 2024 Jul 9;67(7):2696–708. doi: 10.1007/s12033-024-01230-6 (PMC12119671; doi:10.1007/s12033-024-01230-6)
Supplement: Supplementary file 6 — Supplementary file6 (PDF 2264 KB) [file 12033_2024_1230_MOESM6_ESM.pdf]

Intratumoral delivery of genetically engineered anti-IL-6 trans-signaling therapeutics

Online Resource 3

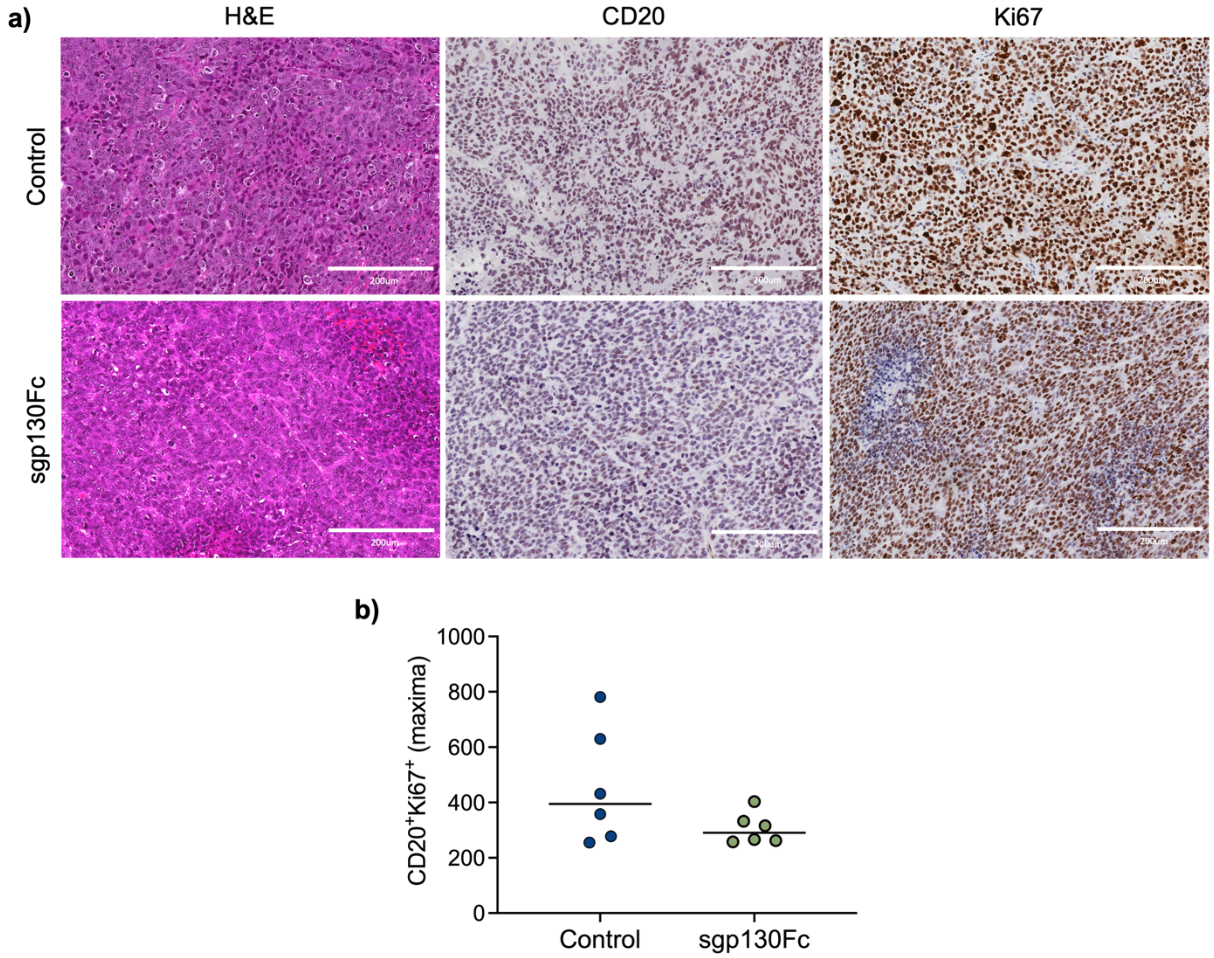

Online Resource 3 a) H&E staining and IHC performed with anti-CD20 and anti-Ki67 antibodies **b)** Double-positive cells ($CD20^{+}Ki67^{+}$) were quantified using ImageJ. Although not statistically significant, control group exhibited higher number of proliferative lymphoma cells
